# The *TNF-α* rs361525 and *IFN-γ* rs2430561 polymorphisms are associated with liver cirrhosis risk: a comprehensive meta-analysis

**DOI:** 10.3389/fimmu.2023.1129767

**Published:** 2023-04-14

**Authors:** Minghui Zheng, Jing Li, Weizhen Fang, Ling Luo, Rui Ding, Hua Zeng, Hong Luo, Xianghua Lin, Chaohui Duan

**Affiliations:** ^1^ Department of Clinical Laboratory, Sun Yat-sen Memorial Hospital of Sun Yat-sen University, Guangzhou, China; ^2^ Guangdong Provincial Key Laboratory of Malignant Tumor Epigenetics and Gene Regulation, Sun Yat-sen Memorial Hospital of Sun Yat-sen University, Guangzhou, China

**Keywords:** tumor necrosis factor-α, interferon-γ, liver cirrhosis, polymorphism, metaanalysis, risk

## Abstract

**Background:**

Inflammation serves as an essential driver of liver cirrhosis (LC) incidence. Accordingly, a meta-analysis was carried out to explore the association between specific polymorphisms in the interferon-γ (*IFN-γ*) and tumor necrosis factor-α (*TNF-α*) genes and the incidence of LC based on comparisons of genotype and allele frequencies.

**Objectives:**

To study the relationship between *TNF-α* rs361525 and *IFN-γ* rs2430561 polymorphisms and the risk of LC.

**Methods:**

A database search was performed for all studies published as of September 10, 2022. The strength of risk relationships was assessed based on odds ratios (ORs) with 95% confidence intervals (CIs).

**Results:**

Pooled analyses were conducted for one common *TNF-α* polymorphism (rs361525) as well as one common *IFN-γ* polymorphism (rs2430561). Both of these SNPs were identified as LC-related risk factors. Specifically, rs361525 was related to LC incidence in both alcoholic liver cirrhosis (OR: 1.86, 95%CI: 1.03-3.34) and hepatitis B virus (HBV)-related cirrhosis cases (OR: 1.44, 95%CI: 1.00-2.06) when using an allelic contrast model. Moreover, rs2430561 was significantly related to LC in an Asian population (OR: 1.45, 95%CI: 1.13-1.86) and in the context of HBV-related cirrhosis (OR: 1.48, 95%CI: 1.13-1.93) when using an allelic contrast model.

**Conclusion:**

These findings indicate that rs361525 and rs2430561 represent LC-related risk factors, although additional large-scale clinical and case-control studies will be vital to confirm these results.

## Introduction

Liver cirrhosis (LC) was responsible for an estimated 2.4% of global mortality in 2017 (1), more than 45% of all deaths in Western nations and over 1.32 million deaths throughout the globe (2).

Many different clinical trials have been developed and implemented in recent years with the goal of defining more effective anti-fibrotic therapies, with phase II and III trials having been performed for a range of agents including obethicolic acid, selonsertib, and elafibranor. While some of these studies have yielded promising findings, in general the benefits have been modest and the outlook for anti-fibrotic treatment remains poor. Accordingly, the most effective strategies currently available to protect against hepatic fibrosis center on its prevention and on alleviating known risk factors associated with this condition (3, 4).

The risk factors known to be most closely related to LC incidence include chronic infections with hepatitis B or C virus (HBV and HCV), which are respectively associated with 39.64 million and 30.36 million cases, as well as nonalcoholic fatty liver disease (NAFLD), which is linked to 10.26 million cases, and alcoholic liver disease (ALD), which is associated with 26.04 million cases. In addition, roughly 16.62 million cases are linked with other underlying causes (5, 6).

Both host genetic factors and immune activity can shape the progression of chronic viral hepatitis and associated infections, making them important determinants of LC risk. Single nucleotide polymorphisms (SNPs) are the most prevalent and extensively explored form of genetic variation (7, 8), and SNPs in genes such as PON1(9), GSTM1(10), and CTLA4(11) have been linked with LC.

Growth factors and cytokines such as members of the interferon (IFN) and tumor necrosis factor (TNF) family are vital components of host immune activity that can shape viral infection-related processes and the subsequent development of fibrosis (12, 13). In a prior study, our group conducted a meta-analysis examining the relationship between mutations in the IL-6 and IL-10 genes (rs1800871, rs1800872, rs1800795, rs1800796, rs1800797, rs1800896) and LC risk, ultimately identifying the IL-10 -592 and -1082 polymorphisms as being closely linked to LC susceptibility(14). Other reports have specifically focused on links between *IFN-γ* and *TNF-α* genes polymorphisms and cirrhotic disease (15).

TNF-α is a potent inflammatory and immunomodulatory cytokine, and the gene that encodes it is associated with the class III MHC region (16). Signaling through the TNF-α/NF-kB axis has been linked to damage, fibrotic activity, and cirrhosis in the liver (17, 18). IFN-γ is another pro-inflammatory cytokine that can influence hematopoietic stem cell development at baseline and serves as a major pathogenic factor associated with various forms of hematopoiesis-related disease stats such as hemophagocytic lymphohistiocytosis, aplastic anemia, and LC (19).

In one recent meta-analysis of 22 studies incorporating 2,638 LC patients and 2,905 control individuals, the *TNF-α* -308A/G polymorphism was found to be unrelated to the risk of developing LC (20). No similar analyses, however, have been performed for the common rs361525 (-238 A>G) polymorphism in this gene or for the common *IFN-γ* rs2430561 polymorphism (+874T/A).

To date, no studies have performed any comprehensive assessments of the *TNF-α* rs361525(21–33) and *IFN-γ* rs2430561(8, 29, 34–40) polymorphisms. Given the above findings and in an effort to overcome limitations associated with small study sample sizes or ethnic/regional variation among study cohorts, the present meta-analysis was thus performed to attempt to more fully clarify the link between these two SNPs in inflammatory cytokine genes and LC risk in an effort to provide a foundation for future clinical reference.

## Materials and methods

### Study selection

In the current study, relevant papers published as of September 10, 2022, were retrieved from PubMed and other databases using the following search terms: “tumor necrosis factor-α or *TNF-α*”, “interferon-γ or *IFN-γ*”, “polymorphism or variation or mutation”, “rs361525”, “rs2430561” and “liver cirrhosis or nonalcoholic fatty liver disease or primary biliary cirrhosis”. This analysis included the research with the largest sample size when several studies used the same set of clinical data.

### Inclusion criteria

Research studies were only considered if they fulfilled the following requirements: (a) studies in which the criteria used to diagnose LC were clearly defined, including liver biopsy, CT, MRI, B-ultrasound, or endoscopic retrograde cholangiopancreatography, (b) studies of correlations between the designated *TNF-α* and *IFN-γ* gene polymorphisms (rs361525 or rs2430561) and LC risk, (c) studies using cohort or case-control designs, (d) studies with enough data to allow for the calculation of 95% confidence interval (95%CI) values, odds ratio (OR), and (e) studies were not duplicated.

### Data extraction

Data extracted from individual studies included first author, country, subject ethnicity, number of cases, year of publication, number of controls, source of control subjects, HWE in the control group, genotyping approach, and LC type. Two investigators (Minghui Zheng, Hong Luo) independently extracted data, with any discrepancies being resolved through discussion and consensus.

### Statistical analysis

Relationships between these *IFN-γ* and *TNF-α* polymorphisms of interest and LC risk were analyzed based on ORs and 95% CIs. Analyzing the significance of ORs was performed using the Z-test (41), while heterogeneity was assessed with the Q-test and *I* ^2^ statistic (42, 43). *I* ^2^ values < 25%, ~50%, and >75% were respectively associated with low, moderate, and high degrees of heterogeneity (44). When heterogeneity was absent, a fixed-effect Mantel-Haenszel model was used *(P*_heterogeneity_ > 0.1), whereas a random-effects DerSimonian-Laird model was instead employed in the presence of heterogeneity (45, 46). Sensitivity analyses were used to assess result stability through a leave-one-out approach (47). Pearson’s chi-square test was used to assess whether polymorphism frequencies diverged from the expected HWE (48). The potential for publication bias was examined through a combination of Begg’s funnel plots and Egger’s regression test (49). Stata 11.0 (StataCorp LP, TX, USA) was used for all statistical analyses.

### Protein-protein interaction network analysis

To better explore links between TNF-α and IFN-γ and LC pathogenesis, Protein-protein interaction networks involving these two genes were generated using the STRING database (http://string-db.org/) (50).

## Results

### Study selection

**Figure 1:**
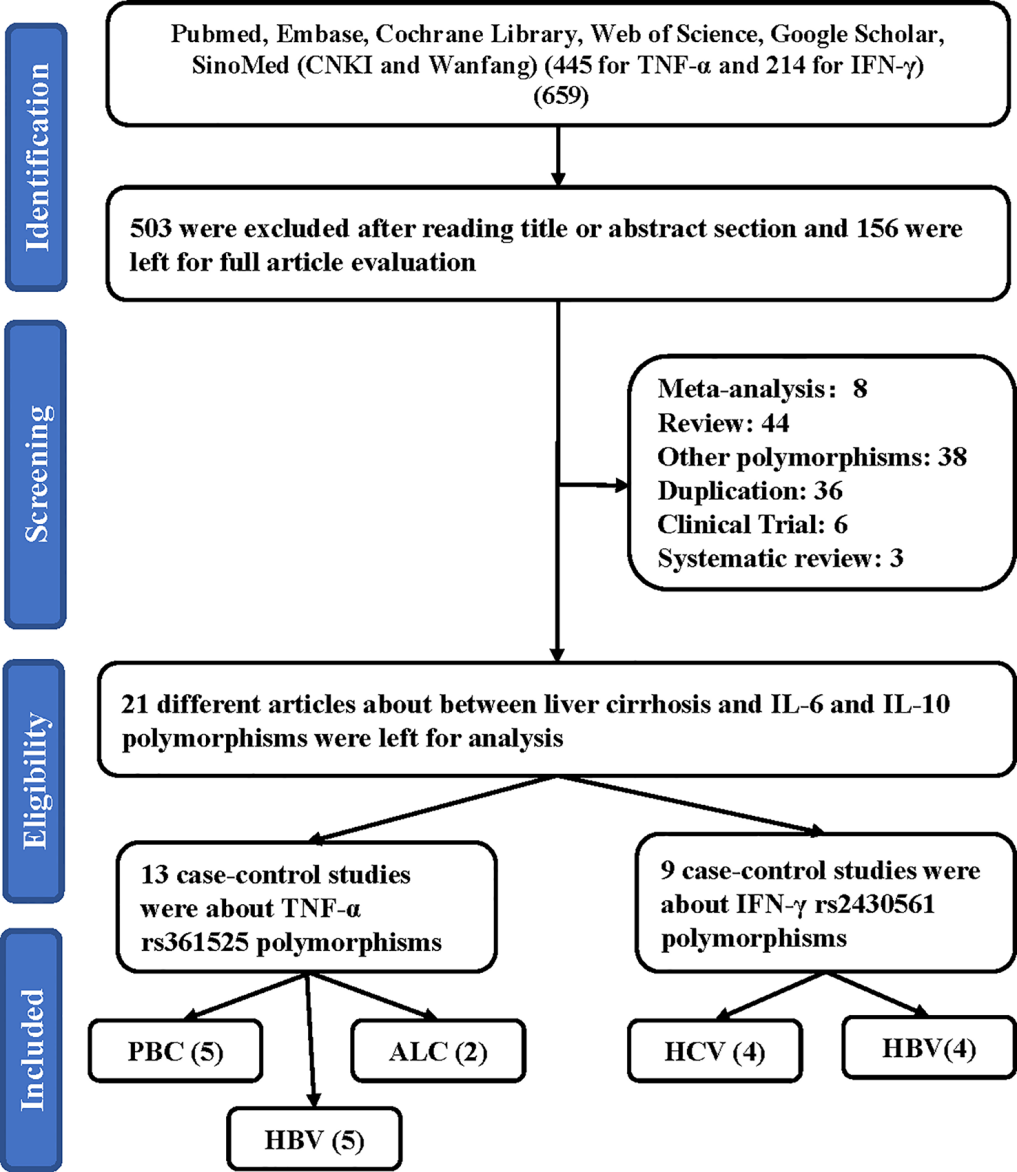
A flowchart outlining the search strategy utilized for the identification of association studies related to the relationship between the *TNF-α* rs361525/*IFN-γ* rs2430561 polymorphisms and the risk of LC.

**Figure 2:**
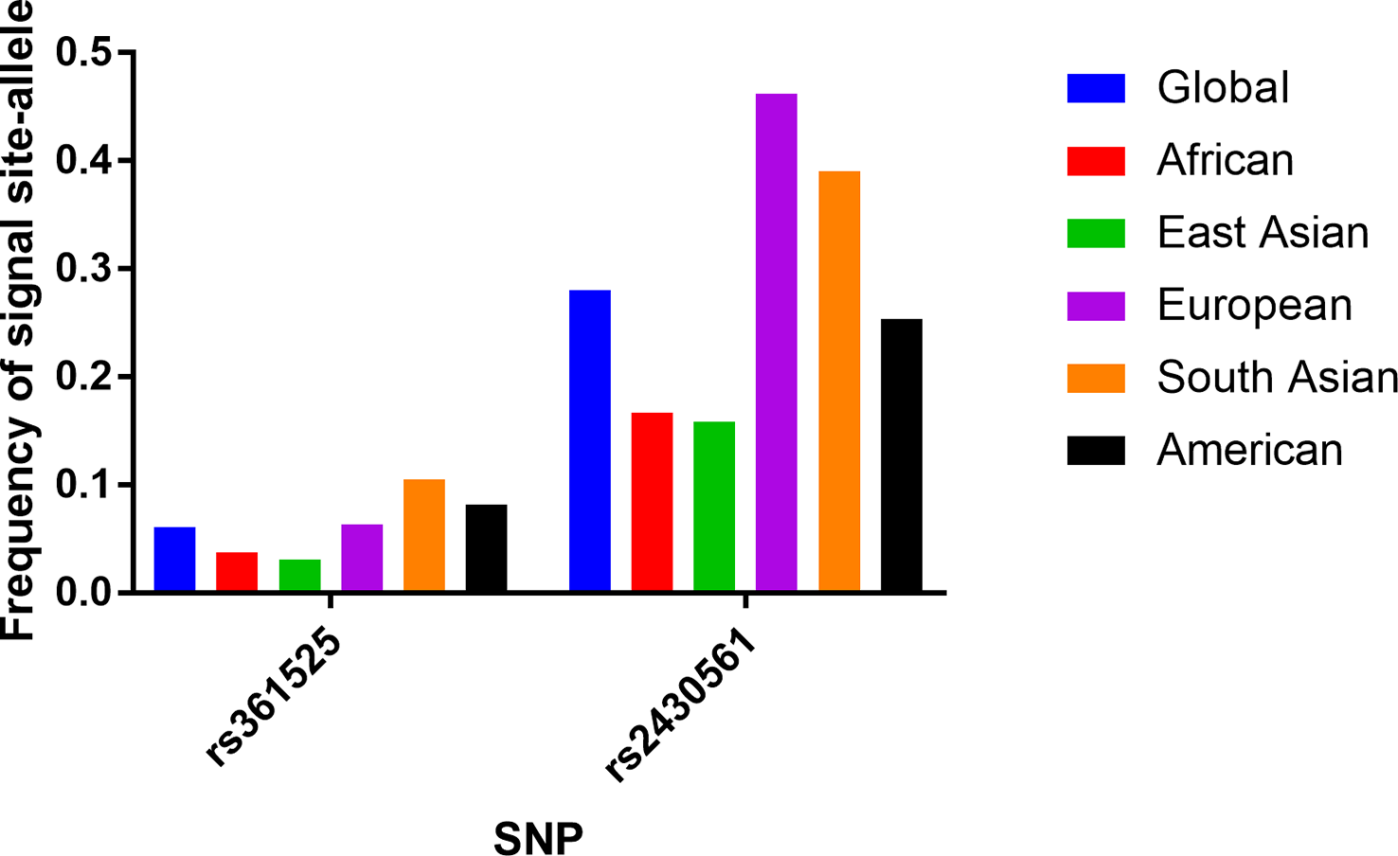
Minor (mutant) allele frequencies for rs361525 and rs2430561 polymorphisms based on data from the online 1000 Genomes database.

Initial literature searches of PubMed and other databases led to the identification of 659 studies (**Figure 1**), including 445 pertaining to the *TNF-α* rs361525 polymorphism and 214 pertaining to the *IFN-γ* rs2430561 polymorphism. Of these studies, 503 were excluded following title and abstract review (no relationship between above two polymorphisms and liver disease), 156 articles were left after reviewing full-text, additional 135 articles were excluded due to following reasons: duplicates (including 36 articles), meta-analyses or systematic reviews (including 11 articles), articles focused on other polymorphisms (including 38 articles), review (including 44 articles), or clinical trials (including 6 articles). The remaining 21 studies (8, 21–40) met with inclusion criteria for this analysis and were thus incorporated into this study, including 13 pertaining to the *TNF-α* rs361525 polymorphism (5, 5, and 2 case-control studies related to PBC, HBV, and ALC, respectively) and 9 pertaining to the *IFN-γ* rs2430561 polymorphism. In all studies, DNA was isolated from samples of patient blood (**Table 1**). Minor allele frequency (MAF) reports for these two SNPs were assessed in 6 different global populations with the 1000 Genomes Browser (https://www.ncbi.nlm.nih.gov/snp/) (**Figure 2**). The data of **Figure 2** is from the online website (https://www.ncbi.nlm.nih.gov/snp/), which is objective and based on statistic data from several genome-wide association studies and published articles. In addition, the difference among different ethnicity may be from ethnicity themselves or other polymorphisms, including other IFN‐γ and TNF‐α polymorphisms.

**Table 1:** Characteristics of included studies about polymorphisms in *TNF-α* and *IFN-γ* genes and cirrhosis of liver risk.

Author	Year	Country	Ethnicity	Case	Control	SOC	HWE	Genotype	Type of liver cirrhosis
rs361525									
Bernal	1999	UK	Caucasian	98	93	PB	0.051	PCR	PBC
Pastor	2005	Spain	Caucasian	65	90	PB	0.534	PCR	ALC
Gordon	1999	UK	Caucasian	91	205	PB	0.706	PCR-RFLP	PBC
Sghaier	2016	Tunisia	African	36	200	PB	0.371	PCR-RFLP	HBV
Osterreicher	2005	Austria	Caucasian	55	94	HB	0.708	PCR-RFLP	HBV/HCV
Nguyen-Khac	2008	France	Caucasian	45	47	PB	0.473	PCR-RFLP	ALC
Niro	2009	Italy	Caucasian	107	141	HB	0.776	PCR-SSP	PBC
Jones	1999	UK	Caucasian	168	145	HB	0.667	PCR	PBC
Zhang	2008	China	Asian	80	61	HB	0.804	AS-PCR	HBV
Li	2014	China	Asian	30	40	HB	0.805	PCR-RFLP	HBV
Fan	2004	China	Asian	57	160	HB	0.763	PCR-RFLP	PBC
Zhang	2013	China	Asian	28	50	HB	0.943	PCR-RFLP	HBV
Ma	2009	China	Asian	163	132	HB	0.009	PCR-RFLP	HBV
rs2430561									
Bahgat	2015	Egypt	African	50	25	HB	0.298	allele-specific PCR	HCV
Sun	2015	China	Asian	126	173	HB	0.087	SSP-PCR	HBV
Wu	2008	China	Asian	32	50	HB	0.276	PCR	HBV
Ma	2009	China	Asian	150	141	HB	0.491	PCR	HBV
Bouzgarrou	2011	Tunisia	African	58	42	HB	0.002	PCR-RFLP	HCV
Sheneef	2017	Egypt	African	50	50	HB	0.004	PCR-RFLP	HCV
Talaat	2022	Egypt	African	69	106	PB	<0.001	SSP-PCR	HCV
Tang	2015	China	Asian	58	56	HB	0.002	PCR-RFLP	HBV
Gao	2010	China	Asian	24	74	PB	<0.001	PCR	HBV/HCV

HB, hospital-based; PB, population-based; SOC, source of control; PCR-RFLP, polymerase chain reaction followed by restriction fragment length polymorphism; SSP, sequence specific primer; AS, allele specific primer; HWE, Hardy-Weinberg equilibrium of control group; PBC, primary biliary cirrhosis; ALC, alcoholic liver cirrhosis; HBV, hepatitis B virus; HCV, hepatitis C virus.

### Quantitative result synthesis

#### *TNF-α* rs361525 polymorphism

**Figure 3:**
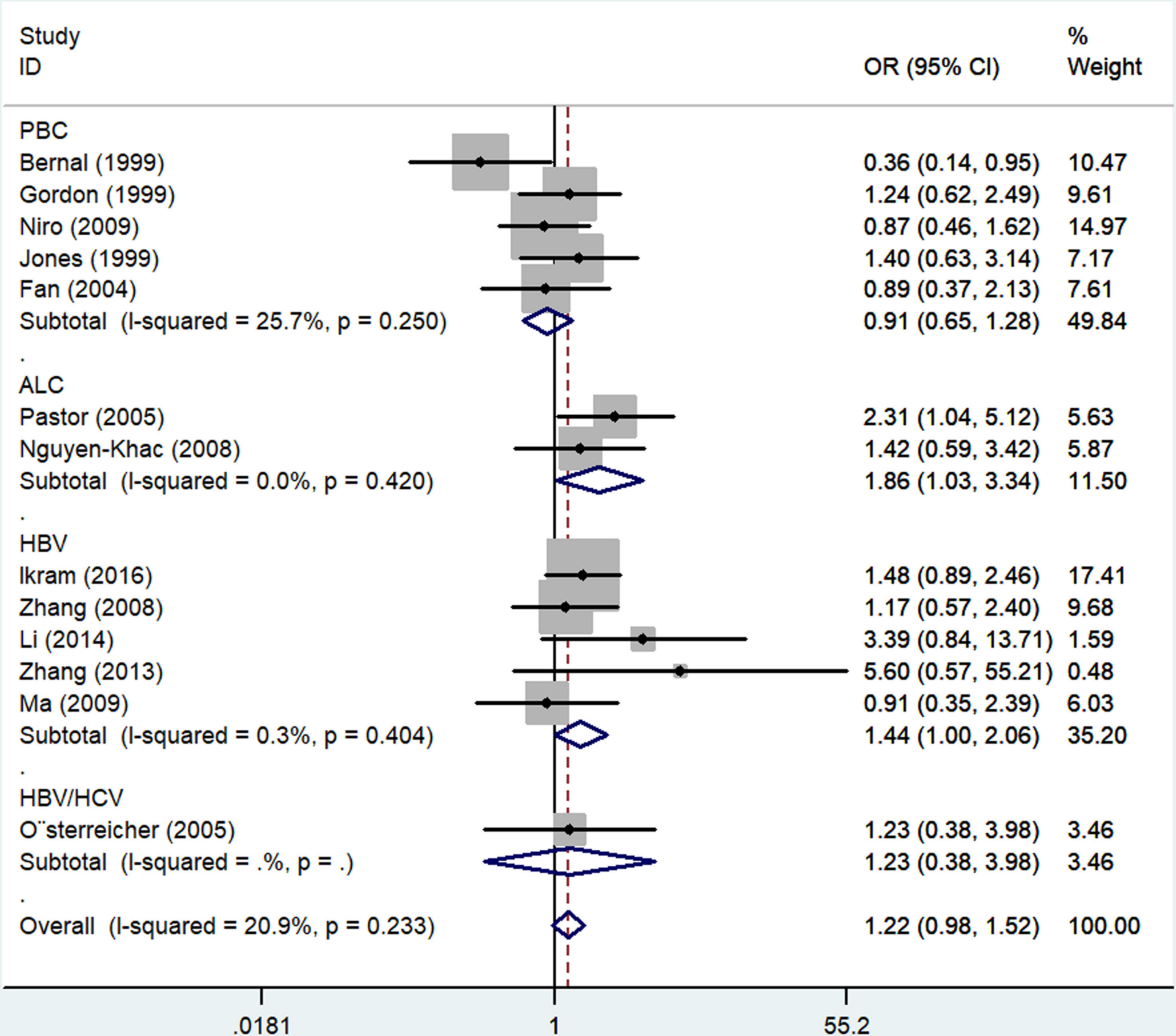
Forest plots depicting the association between rs361525 polymorphisms and LC risk in both the ALC and HBV LC subgroups generated using an allelic contrast model.

In an overall analysis, no significant association with LC risk was detected when using an allelic contrast model (OR: 1.22, 95%CI:0.98-1.52, *P* = 0.233 for heterogeneity, *P* = 0.081, *I* ^2^ = 20.9%). Subgroup analyses also failed to detect any significant association between this polymorphism and LC based on ethnicity or source of control subjects. However, positive relationships between rs361525 and LC risk were detected in the ALC (OR: 1.86, 95%CI:1.03-3.34, *P* = 0.420 for heterogeneity, *P* = 0.040, *I* ^2^ = 7.1%) and HBV (OR: 1.44, 95%CI:1.00-2.06, *P* = 0.404 for heterogeneity, *P* = 0.047, *I* ^2^ = 0.3%) subgroups using allelic contrast models (**Figure 3**; **Table 2**).

**Table 2:** Stratified analyses of *TNF-α* and *IFN-γ* genes’ common polymorphisms on cirrhosis of liver risk.

Variables	N	Case/	Allelic contrast	Heterozygote comparison	Dominant model
		Control	OR(95%CI) *P*_h_ *P I* ^2^	OR(95%CI) *P*_h_ *P I* ^2^	OR(95%CI) *P*_h_ *P I* ^2^
rs361525					
Total	13	1023/1458	1.22(0.98-1.52)0.233 0.081 20.9%	1.14(0.88-1.47)0.392 0.338 5.4%	1.18(0.92-1.52)0.299 0.195 14.5%
Ethnicity					
Asian	5	358/443	1.24(0.80-1.92)0.325 0.326 14.0%	1.38(0.86-2.22)0.379 0.186 4.8%	1.32(0.83-2.10)0.343 0.242 11.0%
Caucasian	7	629/815	1.12(0.83-1.51)0.135 0.441 38.6%	1.01(0.72-1.41)0.272 0.952 0.6%	1.07(0.78-1.47)0.190 0.679 31.2%
SOC					
HB	8	688/823	1.15(0.84-1.58)0.571 0.369 0.0%	1.15(0.82-1.62)0.585 0.423 0.0%	1.16(0.83-1.61)0.583 0.389 0.0%
PB	5	335/635	1.24(0.75-2.04)0.059 0.406 56.0%	1.12(0.75-1.67)0.131 0.594 3.6%	1.19(0.67-2.11)0.080 0.563 51.9%
Disease type					
PBC	5	521/744	0.91(0.65-1.28)0.250 0.592 25.7%	0.85(0.59-1.24)0.454 0.410 0.0%	0.88(061-1.26)0.344 0.490 10.9%
ALC	2	110/137	**1.86(1.03-3.34)0.420 0.040 7.1%**	1.60(0.82-3.14)0.242 0.16827.1%	1.78(0.93-3.39)0.323 0.080 0.0%
HBV	5	337/483	**1.44(1.00-2.06)0.404 0.047 0.3%**	1.50(0.93-2.43)0.465 0.598 0.0%	1.52(0.96-2.41)0.433 0.073 0.0%
rs2430561					
Total	9	617/717	1.03(0.71-1.47)0.000 0.891 73.4%	1.26(0.91-1.76)0.270 0.16419.5%	1.14(0.84-1.54)0.048 0.404 1.8%
Ethnicity					
Asian	5	390/494	**1.45(1.13-1.86)0.197 0.00333.6%**	1.43(0.93-2.18)0.463 0.380 0.0%	**1.57(1.05-2.35)0.046 0.028 0.0%**
African	4	227/223	0.65(0.40-1.07)0.023 0.091 68.5%	1.05(0.62-1.78)0.219 0.86932.2%	0.72(0.45-1.16)0.112 0.173 49.9%
SOC					
HB	7	524/537	0.98(0.60-1.57)0.000 0.920 79.5%	1.26(0.88-1.80)0.410 0.213 1.9%	1.02(0.60-1.73)0.044 0.935 53.7%
PB	2	93/180	1.20(0.84-1.72)0.779 0.326 0.0%	1.07(0.18-6.21)0.050 0.93974.0%	1.42(0.61-3.31)0.111 0.410 60.5%
Disease type					
HCV	4	227/223	0.65(0.40-1.07)0.023 0.091 68.5%	1.05(0.62-1.78)0.219 0.86932.2%	0.72(0.45-1.15)0.112 0.073 49.9%
HBV	4	366/420	**1.48(1.13-1.93)0.116 0.004 9.2%**	**1.61(1.03-2.52)0.694 0.038 0.0%**	**1.73(1.13-2.64)0.718 0.011 0.0%**

P_h_, value of Q-test for heterogeneity test; P, Z-test for the statistical significance of the OR; HB, hospital-based; PB, population-based; SOC, source of control; PBC, primary biliary cirrhosis; ALC, alcoholic liver cirrhosis; HBV, hepatitis B virus; HCV, hepatitis C virus. The bold values stand for the significant relationships.

### *IFN-γ* rs2430561 polymorphism

**Figure 4:**
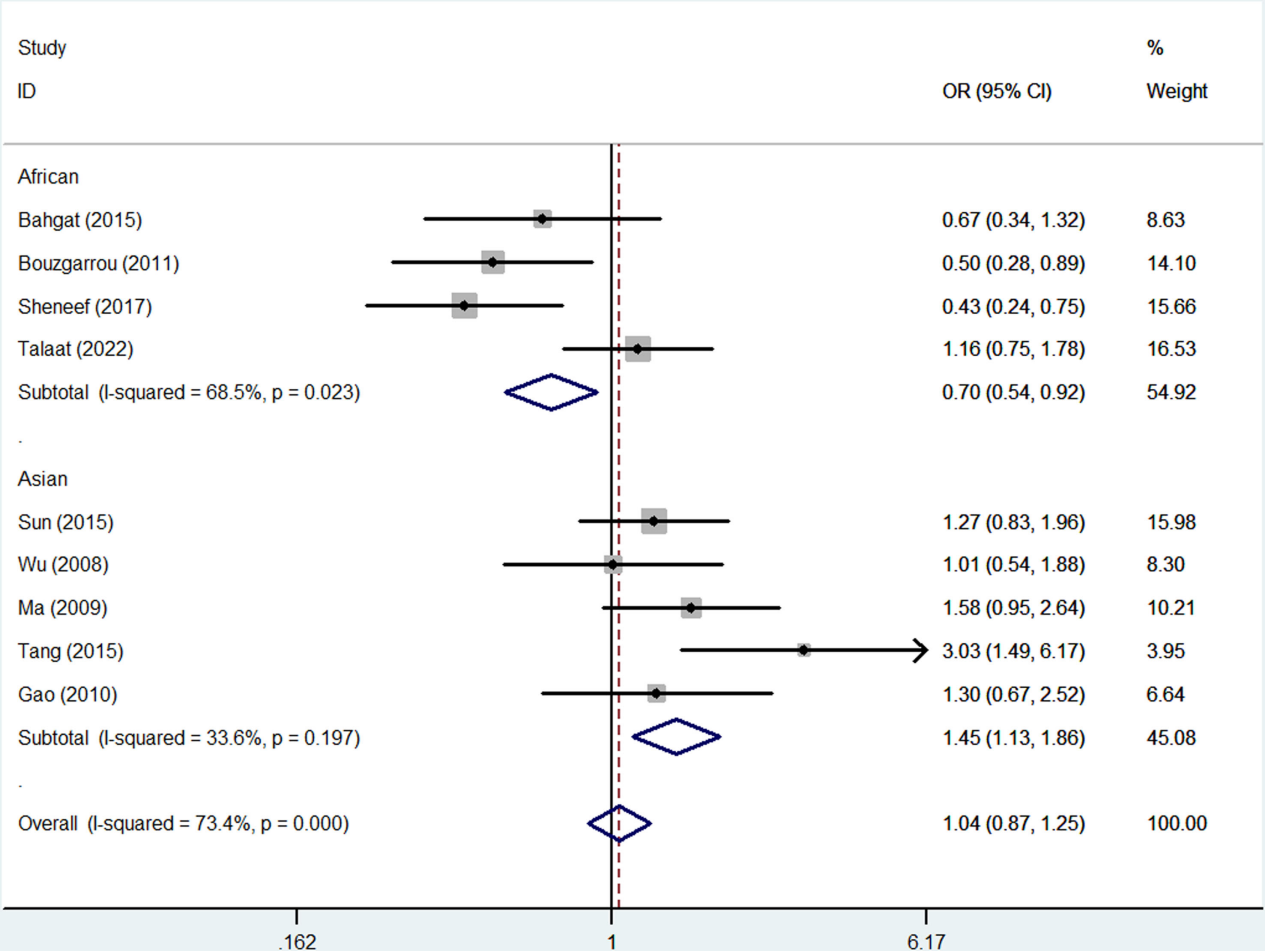
Forest plots corresponding to the association between LC risk and rs2430561 polymorphisms using an allelic contrast model in different ethnic subgroups.

**Figure 5:**
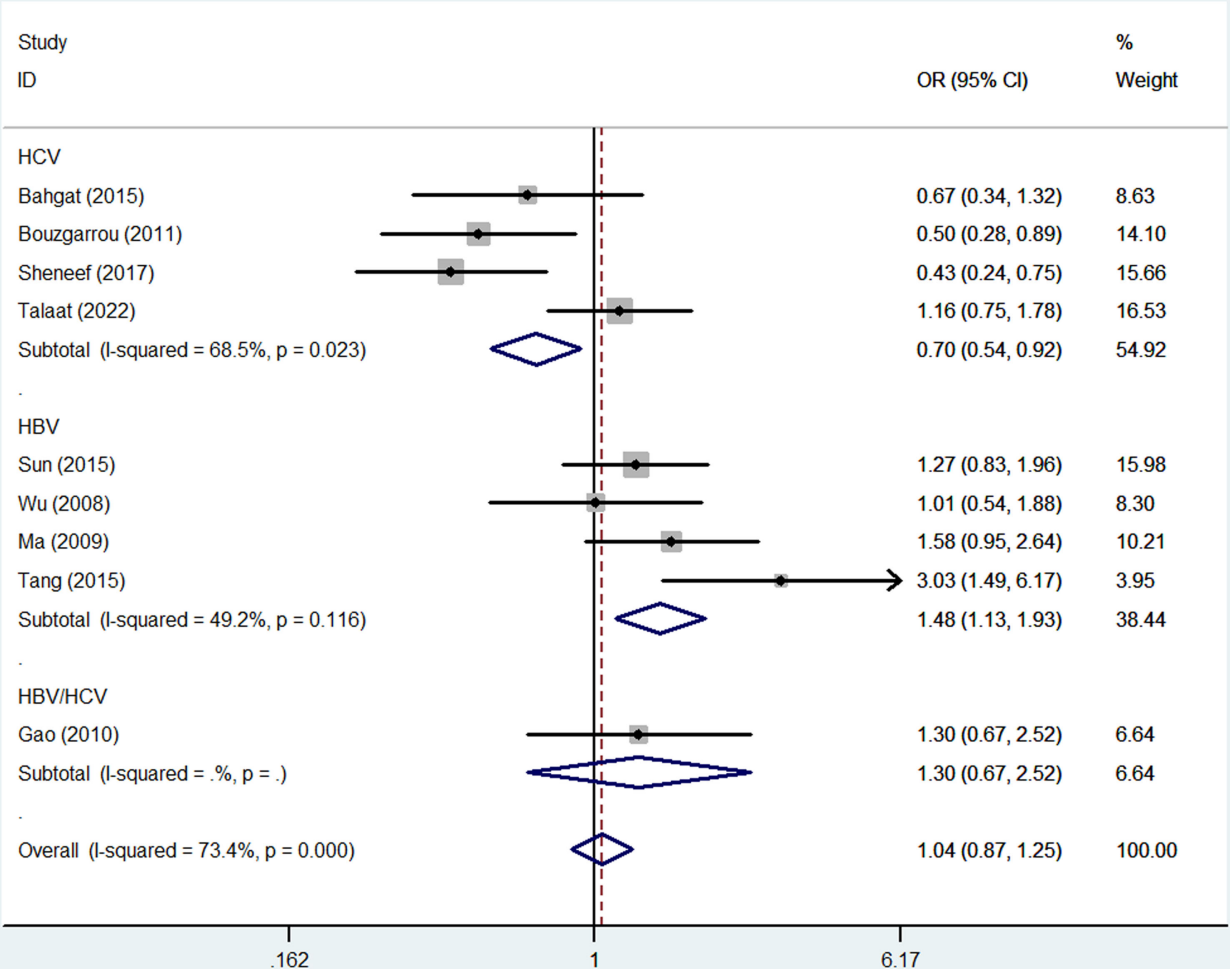
Forest plots corresponding to the association between LC risk and rs2430561 polymorphisms in different LC subgroups generated using an allelic contrast model.

No overall association was detected between rs2430561 and risk of LC, and the same was true when assessing patients based on the source of control subjects. However, this polymorphism was linked to elevated LC risk in Asian individuals (allelic contrast: OR: 1.45, 95%CI:1.13-1.86, *P* = 0.197 for heterogeneity, *P* = 0.003, *I* ^2^ = 33.6%, **Figure 4**; dominant model: OR: 1.57, 95%CI:1.05-2.35, *P* = 0.046 for heterogeneity, *P* = 0.028, *I* ^2^ = 0.0%) and in patients with HBV (allelic contrast: OR: 1.48, 95%CI:1.13-1.93, *P* = 0.116 for heterogeneity, *P* = 0.004, *I* ^2^ = 9.2%, **Figure 5**; heterozygote comparison: OR: 1.61, 95%CI:1.03-2.52, *P* = 0.694 for heterogeneity, *P* = 0.038, *I* ^2^ = 0.0%; dominant model: OR: 1.73, 95%CI:1.13-2.64, *P* = 0.718 for heterogeneity, *P* = 0.011, *I* ^2^ = 0.0%) (**Table 2**).

### Risk of bias and sensitivity analyses

**Figure 6:**
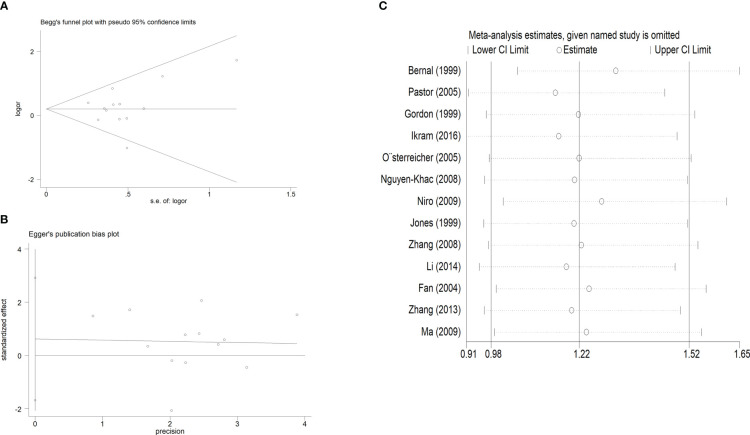
**(A)**. Begg’s funnel plot test for publication bias, with individual points corresponding to specific studies, and the mean effect size being represented by the horizontal axis. Log [OR], natural logaritm of OR. **(B)**. Egger’s publication bias plot. **(C)**: Sensitivity analysis for the association between *TNF-α* rs361525 polymorphisms and LC risk.

**Figure 7:**
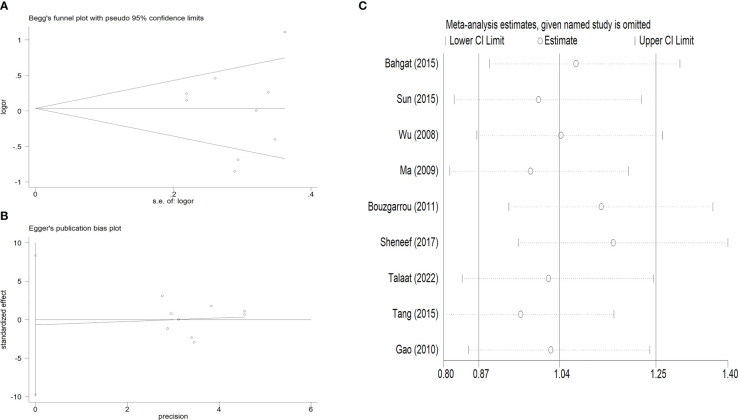
**(A)**. Begg’s funnel plot test for publication bias. **(B)**. Egger’s publication bias plot. **(C)**: Sensitivity analysis for the association between *IFN-γ* rs2430561 polymorphisms and LC risk.

Publication bias was evaluated using Begg’s funnel plots and Egger’s test. Allele-by-allelic comparisons in funnel plots showed asymmetry for these two polymorphisms, which is not attributable to publication bias. When using an allelic contrast model, Egger’s test also showed that there was no publication bias (rs361525: t = 0.6, *P* = 0.564 for Egger’s test; z = 0.18, *P* = 0.855 for Begg’s test; **Figures 6A, B**; rs2430561: t = -0.18, *P* = 0.861 for Egger’s test; z = 0.31, *P* = 0.754 for Begg’s test; **Figures 7A, B**) (**Table 3**).

**Table 3:** Publication bias tests (Begg’s funnel plot and Egger’s test for publication bias test) for *TNF-α* and *IFN-γ* genes polymorphisms.

Egger's test						Begg's test	
Genetic type	Coefficient	Standard error	*t*	*P* value	95%CI of intercept	*z*	*P* value
rs361525							
A-allele vs. G-allele	0.622	1.045	0.6	0.564	(-1.678- 2.923)	0.18	0.855
AG vs. GG	1.239	1.083	1.14	0.277	(-1.146- 3.624)	0.67	0.502
AA+AG vs. GG	1.357	1.125	1.21	0.253	(-1.119- 3.833)	0.67	0.502
rs2430561							
A-allele vs. T-allele	0.692	3.815	-0.18	0.861	(-9.712- 8.328)	0.31	0.754
AT vs. TT	0.709	1.221	-0.58	0.58	(-3.597- 2.179)	1.36	0.175
AA+AT vs. TT	0.409	1.552	-0.26	0.8	(-4.079- 3.261)	0.94	0.348

Additionally, sensitivity analysis was performed to explore factors with the potential to impact the stability of these overall analyses, and none of the included case-control studies were found to alter overall study conclusions under the three tested models for either of the analyzed polymorphisms (**Figures 6C**, **7C**).

### Protein-protein interaction network

**Figure 8:**
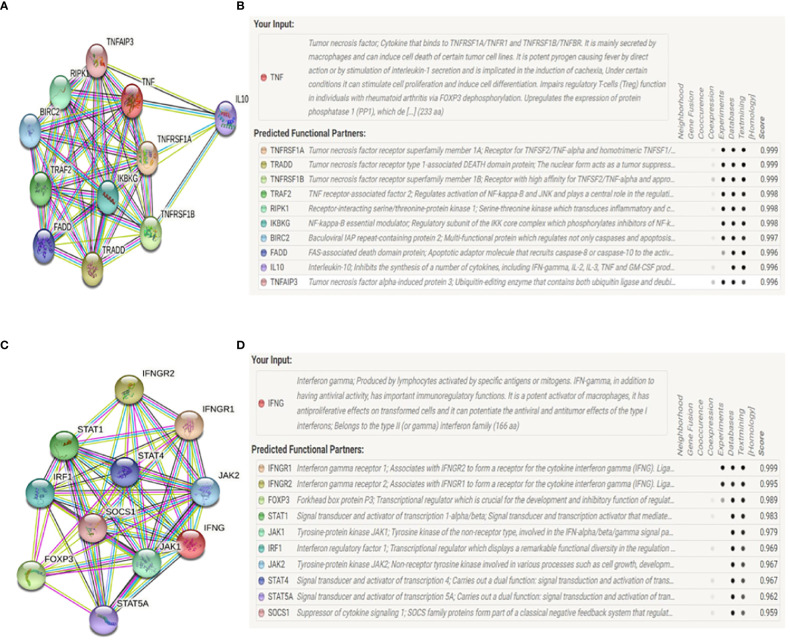
STRING network diagrams depicting interactions between human TNF-α or IFN-γ and other genes. **(A, B)**. At least 10 genes were correlated with: TNFRSF1A: tumor necrosis factor receptor superfamily member 1A; TNFRSF1B: tumor necrosis factor receptor superfamily member 1B; TRADD: tumor necrosis factor receptor type 1-associated DEATH domain protein; TRAF2: TNF receptor-associated factor 2; RIPK1: receptor-interacting serine/threonine-protein kinase 1; FADD: FAS-associated death domine protein; IKBKG: NF-kappa-B essential modulate; BIRC2: baculoviral IAP repeat-containing protein; IL-10: interleukin-10; TNFAIP3: tumor necrosis factor alpha-induced protein 3. **(C, D)**. At least 10 genes were correlated with: IFNGR1: IFN-γ receptor 1; IFNGR2: IFN-γ receptor 2; FOXP3: forkhead box protein P3; JAK1: tyrosine-protein kinase JAK1; JAK2: tyrosine-protein kinase JAK2; IRF1: interferon regulatory factor 1; STAT1: signal transducer and activator of transcription 1-alpha/beta; STAT4: signal transducer and activator of transcription 4; STAT5A: signal transducer and activator of transcription 5A; SOCS1: suppressor of cytokine signaling 1.

Accordingly, the STRING database was used to identify other genes potentially associated with TNF-α or IFN-γ, with these genes then being used to establish putative protein-protein interaction networks (**Figure 8**). The majority of genes identified through this approach were associated with cytokines (TNFRSF1A, TNFRSF1B, TRADD, TRAF2, IL-10, TNFAIP3, IFNGR1, IFNGR2, IRF1, SOCS1), exhibiting interaction scores> 0.9 consistent with very close relationships.

## Discussion

Cirrhosis is the final progressive stage of hepatic fibrosis, and most cases of LC develop in patients with major risk factors such as chronic alcohol abuse or hepatitis caused by viral infections. Histologically, LC is characterized by extensive extracellular matrix material accumulating within the liver parenchyma including very high levels of fibrillar collagen, ultimately compromising the structural integrity and functional utility of this tissue (51).

LC is also closely associated with an elevated risk of hepatocellular carcinoma (HCC), with is the most prominent form of liver cancer in humans, resulting in high rates of morbidity and mortality owing to a lack of effective therapeutic interventions (52). The present study was developed to explore efforts to more effectively diagnose LC and reduce the medical burden associated with this disease by lowering patient mortality rates.

Short−chain fatty acids can influence the production of TNF-α and diverse other inflammatory mediators in response to signaling through the TLR4 pathway, playing important roles in inflammatory and fibrotic activity in the liver through the upregulation of TIMP-1 in activated hepatic stellate cells (HSCs) and through the inhibition of HSC apoptosis (53).

NK-derived IFN-γ can suppress the fibrotic differentiation of HSCs (54), in addition to suppressing proliferative activity, as well as the expression of α-SMA TGF-β (55). In certain contexts, IFN-γ can also promote activated HSC death through mechanisms mediated by the FasL-associated and TRIAL death domains (55). Prolonged HSC activation can contribute to LC incidence.

To date, a range of genes have been linked to the incidence of liver disease including CTLA-4, IL-18, TM6SF2, and GSTM1(10, 56, 57). Inflammation and fibrotic activity are particularly closely associated with LC risk, and various studies have accordingly highlighted possible links between *TNF-α* and *IFN-γ* SNPs and LC incidence. However, many of these studies were based on relatively small sample sizes such that their conclusions were not necessarily robust.

Pastor et al. (26) previously observed a link between the *TNF-α* rs361525 (-238) polymorphism and elevated alcoholic LC risk, while Bouzgarrou et al. (8) similarly observed a higher risk of LC incidence associated with the *IFN-γ* rs2430561 (+874) polymorphism. However, it is critical that these prior studies be combined and that analyses with larger sample sizes be performed to comprehensively examine the link between *TNF-α* and *IFN-γ* SNPs and LC risk.

In this study, in an effort to more fully solidify these relationships between *TNF-α* and *IFN-γ* SNPs and the odds of developing LC, a database search was conducted that ultimately identified 13 and 9 case-control studies respectively associated with the *TNF-α* rs361525 and *IFN-γ* rs2430561 polymorphisms. The pooled analysis of these results ultimately revealed that *TNF-α* rs361525 was linked to LC risk in the ALC and HBV subgroups, while *IFN-γ* rs2430561 was more strongly related to the risk of LC in HBV patients and Asian populations, potentially providing a valuable foundation for future efforts to prevent, diagnose, and treat LC in its earlier stages. This is a study that we need to pay attention to in the future, which is only aimed at determining the polymorphisms (rs361525, rs2430561) of two susceptible genes (TNF-α and IFN-γ) for LC. Based on this conclusion, physical examination of healthy people can be conducted to identify the population with gene mutations, and early intervention, observation, and follow-up can be conducted in such aspects as diet, behavioral habits, and emotional regulation to observe the risk of LC in such people, and further to verify the conclusion in this study, We wish to reduce the incidence rate and the mortality due to disease of LC through this exploration.

LC develops and progresses through a complex multi-factorial process, and the impact of any individual gene on the overall disease process may thus be limited. The STRING online database helps us to find potential interactive genes. The hub gene TNFRSF1A may offer value as a marker of PBC (58), and one GWAS study of 1,840 cases and 5,163 controls identified the TNFRSF1A rs1800693 SNP as a PBC-related risk factor (OR=1.23, 95%CI=1.14-1.33) (59). Peng et al. found TRADD expression to be associated with HBV-related LC and HCC incidence (60), while Lin et al. determined that LC model rats exhibited increased TRAF2 protein content in skeletal muscle samples (61). Moreover, Tan et al. found that RIPK1 acts as a promoter of inflammatory activity in the setting of HCC onset and liver fibrosis (62), and Higuchi et al. reported a link between deleterious TNFAIP3 alleles and autoimmune hepatitis with cirrhosis (63). While Falleti et al. identified two IFNGR1 gene polymorphisms, they failed to find them to be significantly associated with disease (64). In contrast, two IFNGR2 polymorphisms identified by Nalpas et al. that were in strong linkage disequilibrium were found to be closely related to the incidence of liver fibrosis (65). Ramadan et al. determined that reductions in CD4/FoxP3/CD25 levels were evident in patients treated with direct-acting antivirals, contributing to better immunological outcomes in the context of LC (66). There is a strong body of evidence in support of the role of cytokine-induced JAK/STAT signaling serving as a regulator of hepatic fibrosis and regenerative activity (67). Zhu et al. found sennoside A to be capable of inhibiting SOCS1 and decreasing HSC proliferation in liver fibrosis through the inhibition of inflammation (68). Accordingly, additional work should focus on the relationships between these *TNF-α*- and *IFN-γ*-associated genes, gene-gene interactions, and the pathogenesis of LC.

This study is subject to some limitations. For one, additional work focused on African and mixed populations is needed as these groups were underrepresented in studies published to date. In addition, LC is a complex multifactorial disease, and it is thus important that both gene-gene and gene-environment interactions be taken into consideration when examining the etiology of this disease. Particular lifestyle and environmental factors including sex, age, diet, smoking status, family history, history of parasite exposure, immunological factors, and viral infections may all shape the relationship between *TNF-α* or *IFN-γ* polymorphisms and LC. Further work is also necessary to assess whether LC patients face other complications including autoimmunity, hepatitis, abnormal liver function, and HCC incidence. These analyses also did not classify LC stage or pathogenic origin, highlighting important directions for additional study to more precisely guide the prevention and treatment of this disease. Furthermore, we only explored possible interacting genes through online databases, but through literature searches, we have not found that the interactions among 20 genes and the TNF-α or IFN-γ, which is also a topic for future research. Overall, this meta-analysis found some evidence for an association between *TNF-α* rs361525 and *IFN-γ* rs2430561 polymorphisms and the risk of LC suggesting that they may represent viable targets for clinical research aimed at predicting the future risk of severe liver disease. In the future research, we will also try to study a case-control study in our own cohort to confirm current conclusions.

## Data availability statement

The original contributions presented in the study are included in the article/supplementary material. Further inquiries can be directed to the corresponding authors.

## Author contributions

MZ, JL and WF: design research program, collect data, analyze statistics and write articles. LL, RD and HZ: collect data. HL: review the collected data. XL and CD: research design and paper review. All authors contributed to the article and approved the submitted version.
